# High prevalence of the recently identified clonal lineage ST1299/CT3109 *vanA* among vancomycin-resistant *Enterococcus faecium* strains isolated from municipal wastewater

**DOI:** 10.1128/msphere.00396-24

**Published:** 2024-08-27

**Authors:** Giuseppe Valenza, David Eisenberger, Jan Esse, Jürgen Held, Verena Lehner-Reindl, Peter-Louis Plaumann, Tobias Ziegler, Max Knauer, Christian Bogdan, Patrick Dudler

**Affiliations:** 1 Mikrobiologisches Institut—Klinische Mikrobiologie, Immunologie und Hygiene, Universitätsklinikum Erlangen, Friedrich-Alexander-Universität (FAU) Erlangen-Nürnberg, Erlangen, Germany; 2 Bayerisches Landesamt für Gesundheit und Lebensmittelsicherheit, Erlangen, Germany; 3 FAU Profilzentrum Immunmedizin, FAU Erlangen-Nürnberg, Erlangen, Germany; National Institute of Advanced Industrial Science and Technology, Tsukuba, Ibaraki, Japan

**Keywords:** *Enterococcus faecium*, vancomycin-resistance, wastewater, core genome multilocus sequence typing, single nucleotide polymorphisms, ST1299/CT3109, ST117/CT71

## Abstract

Previously, we demonstrated that the majority of vancomycin-resistant *Enterococcus faecium* (VREfm) strains from in-patients of the University Hospital Erlangen, Germany, belonged to only three clonal lineages, namely ST117/CT71 *vanB* and two novel ST1299 *vanA* lineages classified as CT3109 and CT1903. The goal of the current study was (i) to investigate whether VREfm is also detectable in wastewater of the city of Erlangen, (ii) to identify their molecular features, and (iii) to clarify whether VREfm could arise from the community of the city of Erlangen or can be (directly) connected to nosocomial infections in the hospital setting. From April to May 2023, a total of 244 VREfm strains from raw wastewater of the city of Erlangen were analyzed by core genome multilocus sequence typing (cgMLST). Moreover, 20 of them were further investigated for single nucleotide polymorphisms (SNPs). The molecular characterization of the wastewater VREfm strains revealed a high prevalence (27.9%) of the recently identified clonal lineage ST1299/CT3109 *vanA,* which is mainly characterized by the presence of the tetracycline-resistance determinant *tet(M*) and the virulence genes *pilA* and *prpA*. The SNPs analysis revealed the presence of two major clusters, namely cluster I (≤65 SNPs), which included well-known hospital-adapted *vanB* clonal lineages such as ST117/CT71 and ST80/CT1065 and cluster II (≤70 SNPs), which were mainly characterized by the lineage ST1299/CT3109 *vanA*. Based on the concomitant resistance to vancomycin and tetracycline, we propose that ST1299/CT3109 *vanA* primarily originated and spread outside of hospital settings.

**IMPORTANCE:**

This study provides a detailed genomic analysis of vancomycin-resistant *Enterococcus faecium* (VREfm) strains isolated from municipal wastewater with a particular focus on clonal lineages, antimicrobial resistance, and the presence of virulence genes. The high wastewater prevalence of the recently identified clonal lineage ST1299/CT3109 *vanA*, which has been previously detected in hospitals, suggests an enormous potential for future spread in Germany.

## OBSERVATION

*Enterococcus (E.) faecium* is a component of the normal gastrointestinal microbiota of humans and animals. *E. faecium* usually exhibits a low pathogenicity but can cause severe diseases such as bloodstream infections (BSI), endocarditis, and peritonitis (1). Moreover, the treatment of these infections has become difficult due to the emergence of vancomycin-resistant strains (2, 3). Resistance to vancomycin of invasive isolates of *E. faecium* varies substantially among European countries. Germany, with an average resistance rate of 21.6%, belongs to the countries above the European mean value of 17.2% (4).

The population of *E. faecium* is phylogenetically characterized by two main groups, clade A and clade B. Clade A is further subdivided into clades A1 and A2. Most of the human clinical isolates are represented by the hospital-adapted clade A1, which includes sequence types (STs) such as ST117 and ST78, whereas clades A2 and B are associated with farm animals or non-hospitalized human carriers, respectively. Vancomycin resistance is highly prevalent within clade A but is rarely observed among clade B isolates (5).

Antimicrobial resistance and virulence genes play an important role in the selection and spread of microorganisms. Several mechanisms of plasmid-mediated resistance have been described in *E. faecium*, including glycopeptide resistance caused by the presence of *vanA* and *vanB* gene clusters, tetracycline resistance mediated by *tet(M*), and linezolid resistance resulting from the genes *cfr*, *optrA,* or *poxtA* (6). Virulence factors of *E. faecium* are encoded by genes that are localized within genomic pathogenicity islands or in plasmids and commonly split into two main groups: (i) externally secreted factors such as hyaluronidase (*hylEfm*) and secreted antigen A (*sagA*), and (ii) cell-surface antigens like adhesins (*acm, ecbA, prpA, scm*), pili (*pilA, pilB, pilF*), and capsular polysaccharides (*cpsA, cpsB*) (7).

Based on their ubiquity in human and animal feces and persistence in the environment, enterococci have been used as indicators of fecal contamination of water (8). Furthermore, several studies have reported the occurrence of vancomycin-resistant *E. faecium* (VREfm) in hospital and urban wastewater samples with extensive release of hospital-adapted VREfm into the environment (9–17). Among others, Goulioris et al. compared the genomes of VREfm isolated from 20 municipal wastewater treatment plants and from in-patients with BSI of five hospitals in England during the period 2010 to 2016 and found a high genetic relatedness between isolates from a major teaching hospital and nine sewage plants (13).

In a previous study, we analyzed the genetic diversity of 15 invasive VREfm strains isolated from blood cultures of in-patients of the University Hospital Erlangen (UKER), a tertiary-care hospital in Germany with a capacity of approximately 1,400 beds, by core genome multilocus sequence typing (cgMLST) (18). The majority of VREfm isolates (73.3%) belonged to only three clonal lineages: ST117/CT71 *vanB* (*n* = 4), which is the most common vancomycin-resistant lineage in German hospitals, and two novel ST1299 *vanA* lineages classified as CT3109 (*n* = 4) and CT1903 (*n* = 3) that were first detected at the University Hospital Regensburg in the south-eastern part of Germany in 2018 (19, 20).

The aims of the current study were (i) to investigate whether VREfm is also present in wastewater samples of the city of Erlangen; (ii) to identify their molecular features (clonal lineages, antimicrobial resistance, and virulence genes); and (iii) to clarify whether VREfm could arise from the community of the city of Erlangen or can be (directly) connected to nosocomial infections in the hospital setting.

We examined 24 h composite raw wastewater samples from the wastewater treatment plant of the municipal area of Erlangen, which disposes and treats the wastewater of ca. 170,000 inhabitants and ca. 95,000 commercial dischargers. The UKER is located within the served area. During the period April to May 2023, nine wastewater samples were collected in sterile bottles. The isolation of VREfm was carried out by direct plating of 100 µL of each sample on Brilliance VRE Agar (Thermo Fisher Scientific, Basingstoke, UK). After an incubation time of 48 h at 37°C, all colonies of presumptive vancomycin-resistant enterococci were sub-cultured and subsequently analyzed by matrix-assisted laser desorption/ionization time-of-flight technology (Brucker Daltonik GmbH, Bremen, Germany) for identification to species level. The genetic diversity of all VREfm isolates from wastewater was analyzed by cgMLST and *in silico* MLST as previously described (18).

Screening for genetic elements coding for antimicrobial resistance was performed using the databases ResFinder 4.1 (https://cge.cbs.dtu.dk/services/ResFinder/) and AMRFinderPlus (https://www.ncbi.nlm.nih.gov/pathogens/antimicrobial-resistance/AMRFinder/). Screening for putative virulence factors was carried out with the VirulenceFinder 2.0 (https://cge.food.dtu.dk/services/VirulenceFinder/) and the virulence factor database (http://www.mgc.ac.cn/VFs/main.htm). Moreover, selected VREfm strains isolated from wastewater in this study and VREfm strains from hospitalized humans of a previous study (18) were further investigated for single nucleotide polymorphisms (SNPs) using BioNumerics 7.6 software (Applied Maths, Sint-Martens-Latem, Belgium). The following criteria were applied for the selection of the wastewater strains: (i) belonging to the five most frequently detected clonal lineages, (ii) the number of isolates per clonal lineage according to the proportion of detected clonal lineages, and (iii) no more than one isolate per clonal lineage from each sample collection.

Antimicrobial susceptibility testing was carried out using VITEK 2 AST-P616 cards (bioMérieux, Marcy-l'Étoile, France) and MIC Test Strips (Liofil chem srl, Roseto degli Abruzzi, Italy). The results were interpreted according to (i) the European Committee on Antimicrobial Susceptibility Testing breakpoints (https://www.eucast.org/clinical_breakpoints) for ampicillin, ampicillin/sulbactam, imipenem, ciprofloxacin, erythromycin, vancomycin, teicoplanin, and linezolid, and (ii) the Clinical and Laboratory Standards Institute breakpoints for tetracycline and daptomycin (https://view.officeapps.live.com/op/view.aspx?src=https%3A%2F%2Fclsi.org%2Fmedia%2F0fcjerwm%2Fpart_b_clsi_vs_fda-breakpoints.xlsx&wdOrigin = BROWSELINK).

All statistical analyses were performed using Stata SE (version 16.1).

During the period April to May 2023, 244 VREfm strains from raw wastewater of the city of Erlangen were included in this study. Tables 1 and 2 provide a summary of all sequence types and the molecular characteristics of the strains. *In silico* MLST revealed that the strains belonged to five different well-known STs, namely ST117 (*n* = 88), ST1299 (*n* = 75), ST80 (*n* = 40), ST721 (*n* = 30), ST18 (*n* = 9), and one new ST, whose number has not yet been assigned (*n* = 2). The majority of the strains (81.9%) were associated with five clonal lineages: ST1299/CT3109 *vanA* (*n* = 68; 27.9%), ST117/CT71 *vanB* (*n* = 53; 21.7%), ST721/CT6962 *vanB* (*n* = 30; 12.3%), ST80/CT1065 *vanB* (*n* = 30; 12.3%), and ST117/CT929 *vanA* (*n* = 19; 7.8%).

**TABLE 1:** Distribution of vancomycin-resistant *E. faecium* sequence types from wastewater during the sampling period

Date of sampling	Sequence types (ST)					
	ST18 (*n*)	ST80 (*n*)	ST117 (*n*)	ST721 (*n*)	ST1299 (*n*)	n.a.^*a*^ (*n*)
18.04.2023	3	7	4	0	12	2
25.04.2023	1	0	10	15	7	0
27.04.2023	5	4	2	1	1	0
02.05.2023	0	1	13	0	18	0
04.05.2023	0	4	11	4	3	0
09.05.2023	0	8	17	4	9	0
11.05.2023	0	3	5	0	4	0
16.05.2023	0	10	19	3	6	0
23.05.2023	0	3	7	3	15	0
Total	9	40	88	30	75	2

^*a*^ New sequence type, ST number not yet assigned.

**TABLE 2:** Molecular characteristics of vancomycin-resistant *E. faecium* strains from wastewater (*n* = 244)

STs	ST18	ST80							ST117						ST721	ST1299		n.a.^*a*^
Number of isolates	9	40							88						30	75		2
CTs	7597	1065	2680	3356	6820	7665	7677	7930	71	929	2505	5130	6681	7134	6962	1903	3109	7133
Number of isolates	9	30	1	1	1	1	4	2	54	19	7	3	4	1	30	6	69	2
Antimicrobial resistance genes (*n*)																		
*vanA* (109)	0	0	1	1	1	0	0	2	0	19	7	0	4	0	0	6	68	0
*vanB* (133)	9	30	0	0	0	0	4	0	53	0	0	3	0	1	30	0	1	2
*vanA+vanB* (2)	0	0	0	0	0	1	0	0	1	0	0	0	0	0	0	0	0	0
*tet(M)* (78)	0	1	0	0	1	0	0	2	0	0	4	0	0	0	0	3	67	0
*cfr* (0)	0	0	0	0	0	0	0	0	0	0	0	0	0	0	0	0	0	0
*optrA* (0)	0	0	0	0	0	0	0	0	0	0	0	0	0	0	0	0	0	0
*poxtA* (0)	0	0	0	0	0	0	0	0	0	0	0	0	0	0	0	0	0	0
Virulence genes (*n*)																		
*acm* (244)	9	30	1	1	1	1	4	2	54	19	7	3	4	1	30	6	69	2
*bopD* (244)	9	30	1	1	1	1	4	2	54	19	7	3	4	1	30	6	69	2
*capD* (167)	9	29	1	0	0	1	4	2	54	19	7	3	4	1	30	0	1	2
*cpsA* (244)	9	30	1	1	1	1	4	2	54	19	7	3	4	1	30	6	69	2
*cpsB* (244)	9	30	1	1	1	1	4	2	54	19	7	3	4	1	30	6	69	2
*dll* (244)	9	30	1	1	1	1	4	2	54	19	7	3	4	1	30	6	69	2
*ecbA* (167)	9	30	0	0	0	1	4	2	54	19	7	3	4	1	30	0	1	2
*efaAfm* (244)	9	30	1	1	1	1	4	2	54	19	7	3	4	1	30	6	69	2
*hylEfm* (216)	9	30	0	1	1	1	4	2	54	19	7	0	4	1	30	0	51	2
*fruA* (244)	9	30	1	1	1	1	4	2	54	19	7	3	4	1	30	6	69	2
*pilA* (93)	0	0	1	1	1	1	0	2	0	19	7	3	4	0	0	6	48	0
*pilB* (244)	9	30	1	1	1	1	4	2	54	19	7	3	4	1	30	6	69	2
*pilF* (35)	0	0	0	1	0	0	0	2	0	18	7	3	4	0	0	0	0	0
*prpA* (73)	0	0	0	0	0	0	0	1	0	12	6	2	2	0	0	5	45	0
*sagA* (243)	9	30	1	1	1	1	4	2	54	19	7	3	4	1	29	6	69	2
*scm* (139)	9	24	1	0	1	1	3	2	44	14	4	2	4	0	26	0	2	2
*sgrA* (242)	9	29	1	1	1	1	4	2	54	19	7	3	4	0	30	6	69	2
*srtC* (244)	9	30	1	1	1	1	4	2	54	19	7	3	4	1	30	6	69	2

^*a*^ New sequence type, ST number not yet assigned.

With regard to the antimicrobial resistance determinants, 133 strains (54.5%) harbored the *vanB*- and 109 (44.7%) the *vanA*-gene. Moreover, two strains (0.8%) simultaneously carried both genes. The tetracycline-resistance determinant *tet(M*) was detected in 78 strains and occurred mainly among the strains belonging to the lineage ST1299/CT3109 *vanA* (67/68). In addition, none of the strains showed genes coding for linezolid resistance. With regard to the virulence factors genes, all strains harbored *acm, bopD, cpsA, cpsB, dll, efaAfm, fruA, pilB,* and *srtC*. Among the wastewater strains belonging to the two most frequently detected clonal lineages of this study, the virulence genes *capD, ecbA,* and *scm* were identified more frequently in the ST117/CT71 *vanB* strains. In contrast, *pilA* and *prpA* exclusively occurred in the ST1299/CT3109 *vanA* strains (*P* < 0.05).

**Fig 1:**
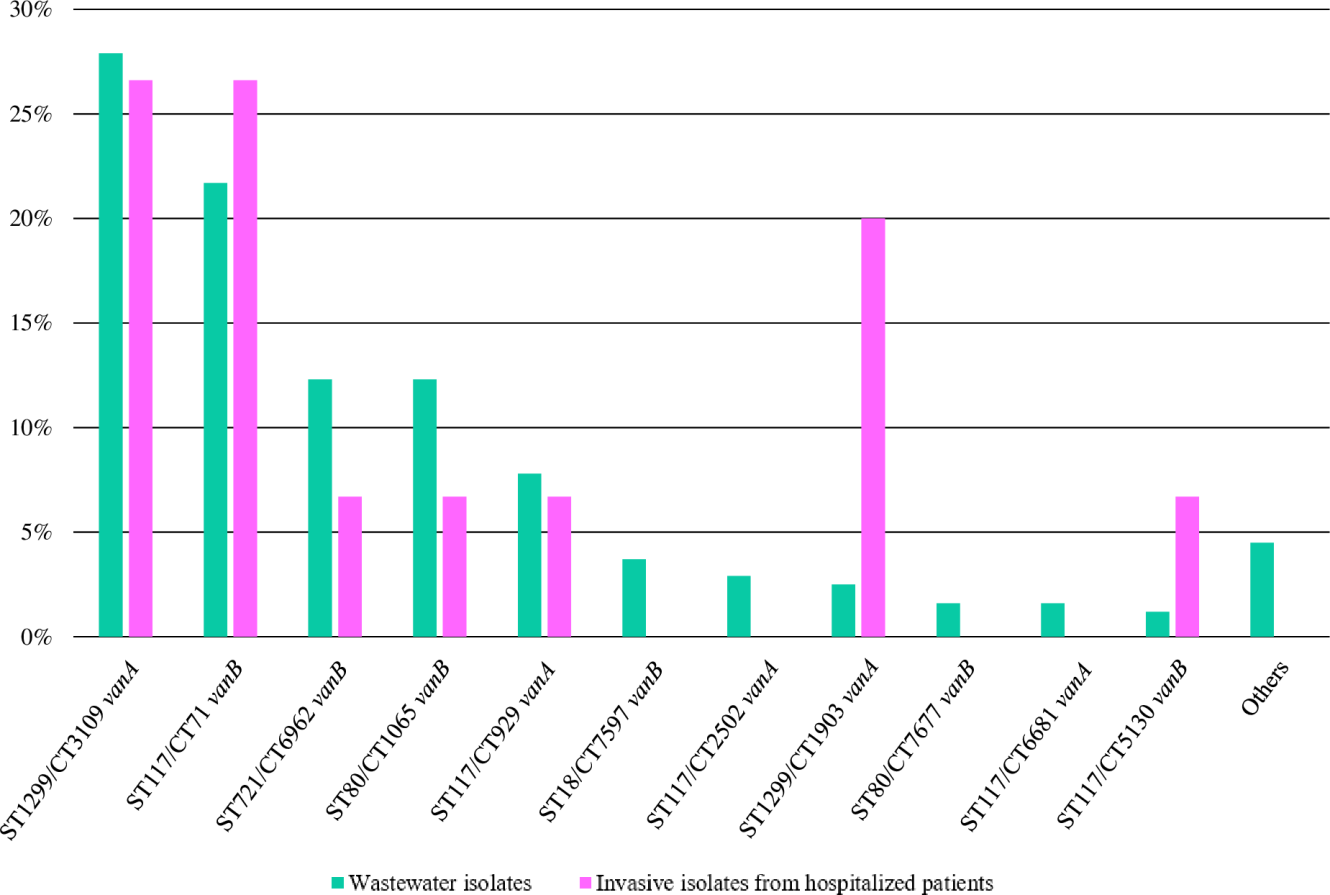
Frequency of the clonal lineages in vancomycin-resistant *E. faecium* strains isolated from wastewater (*n* = 244; this study) and from in-patients of the University Hospital Erlangen [*n* = 15; previous study (18)].

A comparison between the VREfm strains from wastewater samples of this study (*n* = 244) and the VREfm strains from in-patients of UKER (*n* = 15) of a previous study (18) showed the predominance of the clonal lineages ST1299/CT3109 *vanA* and ST117/CT71 *vanB* in both populations. Furthermore, all clonal lineages detected at UKER also occurred in wastewater of the city of Erlangen (Fig. 1).

**Fig 2:**
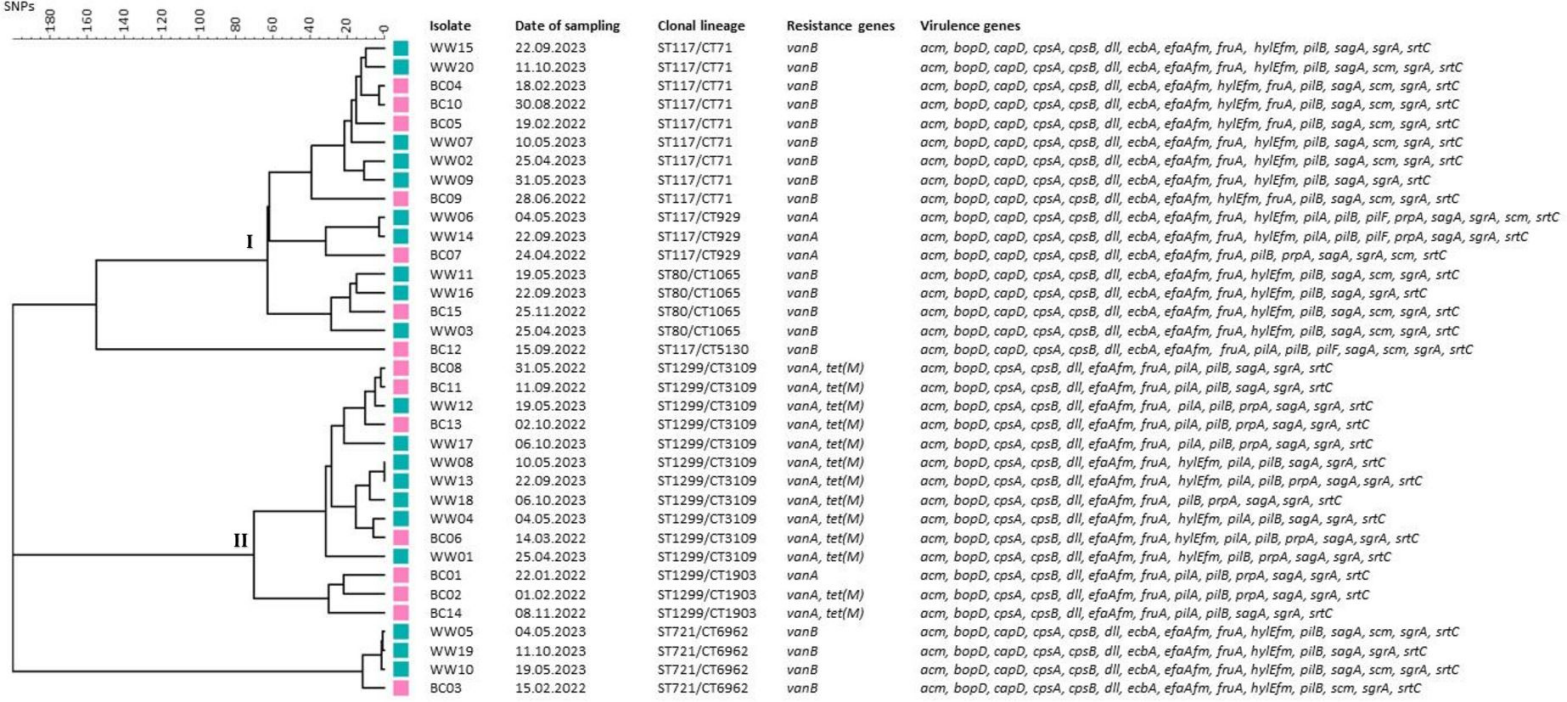
Dendrogram of the genetic diversity of selected vancomycin-resistant *E. faecium* strains isolated from wastewater (green, *n* = 20; this study) and from in-patients of the University Hospital Erlangen [pink, *n* = 15; previous study (18)] after SNP-analysis.

The SNPs-analyses of 20 selected strains from wastewater and 15 strains from in-patients of UKER revealed the presence of two major clusters, namely cluster I (≤65 SNPs), which includes hospital-adapted *vanB* clonal lineages such as ST117/CT71 and ST80/CT1065 and cluster II (≤70 SNPs), which is mainly represented by the lineage ST1299/CT3109 *vanA*. Moreover, strains from wastewater and hospitalized patients belonging to the same clonal lineages showed high similarity (mostly <20 SNPs) (Fig. 2).

Antimicrobial susceptibility testing of the selected wastewater strains (*n* = 20) confirmed vancomycin resistance in all strains, whereas the teicoplanin resistance rate amounted to 45%. In addition, all strains were also resistant to ampicillin, ampicillin/sulbactam, imipenem, ciprofloxacin, and erythromycin. Interestingly, all strains that showed a phenotypic tetracycline resistance (*n* = 7; 35%) harbored the gene *tet(M*) and belonged to the clonal lineage ST1299/CT3109 *vanA*. Moreover, none of the 20 strains was resistant to linezolid or daptomycin.

As the expected number of SNPs to accumulate in an *E. faecium* core genome during 6-months period ranges from 1 to 29 (21), it cannot be excluded that most of the wastewater VREfm strains have a nosocomial origin as previously described (9–17). However, strains of the lineage ST1299/CT3109 *vanA* differ from other strains by the presence of the *tet(M*) gene, which confers resistance to tetracycline. Among the VREfm-strains from in-patients of UKER, all isolates belonging to ST1299/CT3109 *vanA* (BC06, BC8, BC11, BC13) carried *tet(M*). In contrast, none of the isolates belonging to the most common vancomycin-resistant lineage in German hospitals, ST117/CT71 *vanB*, (BC04, BC05, BC09, BC10) harbored tet(M). Moreover, among the VREfm-strains from wastewater of this study, 67 of 68 isolates belonging to ST1299/CT3109 *vanA* carried *tet(M*). In contrast, none of the 53 ST117/CT71 *vanB* isolates harbored *tet(M)* (Fig. 2; Table 2). Based on these findings, it can be assumed that *tet(M*) and *vanA* are located in the same mobile genetic element (e.g., plasmid or transposon) of ST1299/CT3109 strains. The above-mentioned genetic linkage could play a role in the selection and spread of ST1299/CT3109 because tetracycline is frequently prescribed in the community, but only rarely used for patients treated in hospitals. A recent study about the prevalence of nosocomial infections and the use of antimicrobials in German hospitals revealed that tetracycline was not among the 10 most widely applied antimicrobials, which were piperacillin/tazobactam, ampicillin/sulbactam, cefuroxime, ceftriaxone, meropenem, cefazolin, amoxicillin/clavulanic acid, metronidazole, clindamycin, and vancomycin (22). In contrast, tetracyclines ranked at position 4 of the antibiotics used for the treatment of outpatients after penicillins, cephalosporins, and macrolides (23) and represented the second most commonly administered substance group after penicillins for the anti-infective therapy of pets and farm animals (24). Based on these premises, it is tempting to speculate that ST1299/CT3109 *vanA* primarily originated from and spread within the outpatient sector and/or the veterinary field of application. As human feces affect municipal wastewater composition substantially, the outpatient sector is the most likely origin of ST1299/CT3109 *vanA*. However, a contribution by the animal and food sector (e.g., slaughterhouses, meat processing facilities, or pet-derived human colonizations) cannot be ruled out depending on local conditions.

In conclusion, this study revealed a high prevalence of the recently identified clonal lineage ST1299/CT3109 *vanA* among VREfm strains isolated from municipal wastewater. This lineage is mainly characterized by the presence of the tetracycline-resistance determinant *tet(M*) and the virulence genes *pilA* and *prpA*. Based on the concomitant resistance to vancomycin and tetracycline in the above-mentioned strains, we propose that ST1299/CT3109 *vanA* originated and spread primarily in the community outside of hospitals.

## Data Availability

The raw sequencing data were published at the European Nucleotide Archive (ENA), project number PRJEB72879.

## SUPPLEMENTAL MATERIAL

Table S1 msphere.00396-24-s0001.xlsx Metadata of all VREfm strains.

Table S2 msphere.00396-24-s0002.xlsx Antimicrobial susceptibility testing results.

ASM does not own the copyrights to Supplemental Material that may be linked to, or accessed through, an article. The authors have granted ASM a non-exclusive, world-wide license to publish the Supplemental Material files. Please contact the corresponding author directly for reuse.
